# Corrosion Study of Mild Steel in Aqueous Sulfuric Acid Solution Using 4-Methyl-4H-1,2,4-Triazole-3-Thiol and 2-Mercaptonicotinic Acid—An Experimental and Theoretical Study

**DOI:** 10.3389/fchem.2017.00061

**Published:** 2017-08-24

**Authors:** Valbonë V. Mehmeti, Avni R. Berisha

**Affiliations:** Department of Chemistry, Faculty of Natural and Mathematic Sciences, University of Prishtina Pristina, Serbia

**Keywords:** mild steel, corrosion inhibitors, quantum methods, mercapto compounds

## Abstract

The corrosion behavior of mild steel in 0.1 M aqueous sulfuric acid medium has been studied using weight loss, potentiodynamic polarization measurements, quantum chemical calculations, and molecular dynamic simulations in the presence and absence of 4-methyl-4H-1,2,4-triazole-3-thiol and 2-mercaptonicotinic acid. Potentiodynamic measurements indicate that these compounds mostly act as mixed inhibitors due to their adsorption on the mild steel surface. The goal of the study was to use theoretical calculations to better understand the inhibition. Monte Carlo simulation was used to calculate the adsorption behavior of the studied molecules onto Fe (1 1 1) and Fe_2_O_3_ (1 1 1) surface. The molecules were also studied with the density functional theory (DFT), using the B3LYP functional in order to determine the relationship between the molecular structure and the corrosion inhibition behavior. More accurate adsorption energies between the studied molecules and iron or iron oxide were calculated by using DFT with periodic boundary conditions. The calculated theoretical parameters gave important assistance into the understanding the corrosion inhibition mechanism expressed by the molecules and are in full agreement with the experimental results.

## Introduction

Mild steel is a valuable construction material used in myriad diverse industries mostly for to its outstanding mechanical properties and its almost insignificant cost compared to other materials (Al-Amiery et al., 2014; Su et al., 2016). However, mild steel as many other metals is prone to corrosion, hence their surface must be protected from this undesired process (Migahed et al., 2004). The protection of metals, apart from the use of classic inhibitors (Sanyal, 1981; Selvi et al., 2003; Fouda and Ellithy, 2009; Obi-Egbedi et al., 2011b; Finšgar and Jackson, 2014; Berisha et al., 2015b; Mohsenifar et al., 2016), might be achieved by chemical or electrochemical surface modification such as, SAM's (Self-Assembled Monolayers) formed from silanes (Palanivel et al., 2005; Van Ooij et al., 2005), phosphonic acids (Abohalkuma and Telegdi, 2015; Kosian et al., 2016), sodium oleate (Shubha et al., 2013), or electrochemical reduction of aryldiazonium salts on metals (Chaussé et al., 2002; Berisha et al., 2011, 2015a). Nearly all of the important acid inhibitors are organic molecules containing heteroatoms like nitrogen, oxygen, phosphorous, sulfur, etc. It has been claimed that the corrosion protection performance exhibited by these molecules decreases following the order: O > N > S > P (Berisha et al., 2015b; Mohsenifar et al., 2016). As triazole derivatives represent a widely known class of molecules that exhibit very good corrosion inhibition properties toward mild steel we selected one of such molecule for our study (Bentiss et al., 1999; Selvi et al., 2003; Döner et al., 2011). Density functional theory has become a convenient method to decipher experimental results, permitting to obtain reliable structural parameters for molecules (Geerlings et al., 2003; Gece, 2008; Berisha et al., 2015b). In corrosion studies, this method makes it possible to accurately predict the inhibition efficiency of organic corrosion inhibitors on the basis of electronic and molecular properties as well as reactivity indexes (Obot et al., 2015). In this study, two different mercapto compounds: (a) 2-mercaptonicotinic acid (2MA) and (b) 4-methyl-4H-1,2,4-triazole-3-thiol (4MT) were used as corrosion inhibitors of mild steel sulfuric acid solution (*c* = 0.1 M). The adsorption mechanism and inhibition performance of 4MT and 2MA molecules (in the neutral and protonated forms) acid were examined as corrosion inhibitors by means of DFT at the B3LYP/6-31G (d,p) basis set level. Moreover, molecular dynamics simulations where used to calculate the adsorption geometries of the adsorbate and DFT with a plane wave basis set calculations to evaluate more precisely adsorption energies and the interaction with the iron or iron oxide surface.

**Graphical Abstract:**
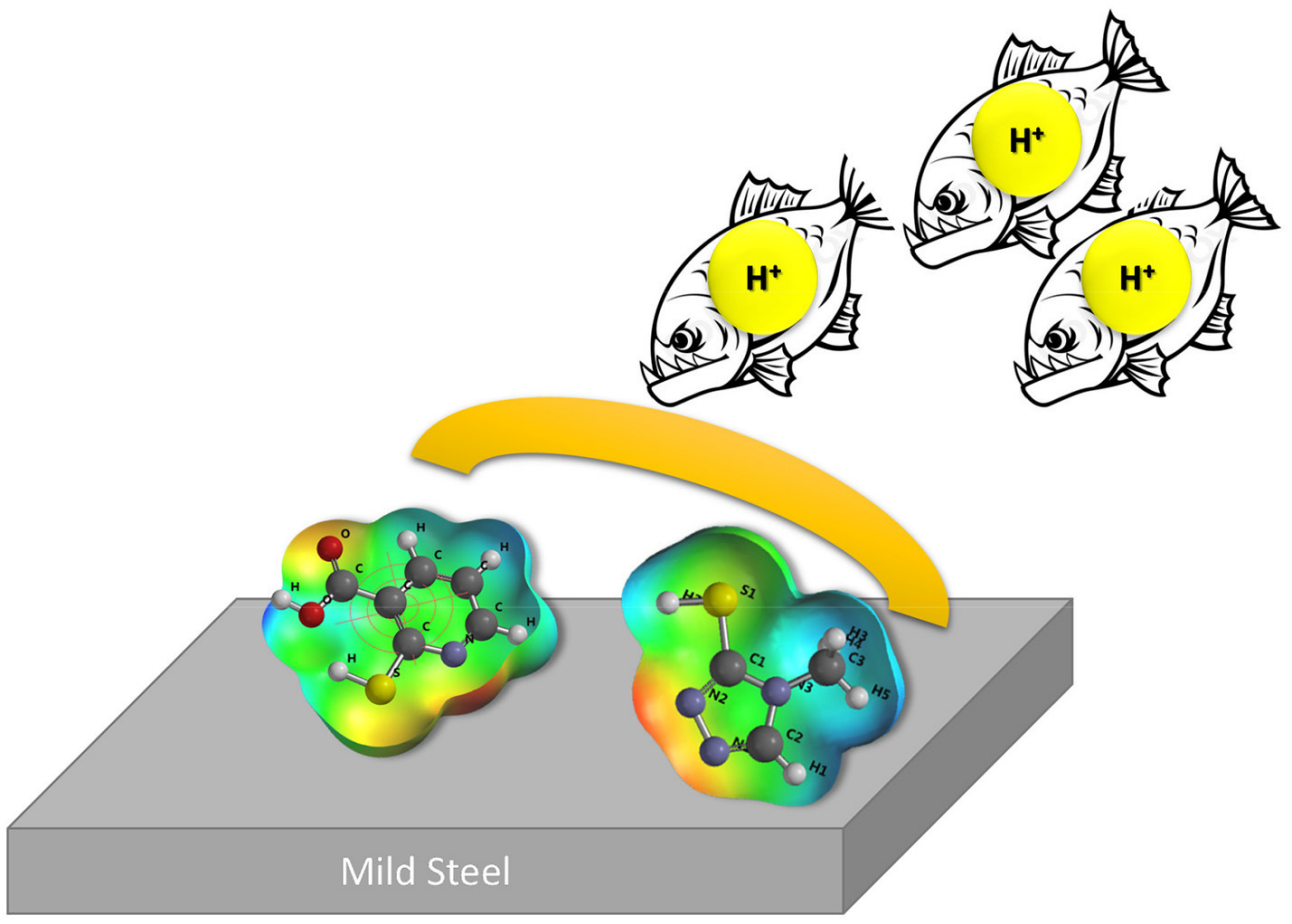
Adsorption of 2MA and 4MT acts as proton barrier that decreases the corrosion rate of mild steel in the acidic aqueous media.

### Experimental

For the electrochemical measurements, the electrode was prepared by embedding a mild steel wire (*d* = 2 mm, *l* = 10 mm) inside a Teflon® (*d* = 1 cm, *l* = 6 cm) tube with epoxy resin. Prior to its use, the electrode was polished on silicon carbide abrasive paper (medium grain diameter 6.5–15.3 microns), then on a (DP-Nap) cloth with an aluminum oxide (0.3 micron particle size) suspension, then the electrode was washed and sonicated in water. The chemical composition of the electrode and the coupons was as follows: iron 99.5494%, carbon 0.1252%, phosphorous 0.0316%, manganese 0.1831%, silicon 0.0561%, chromium 0.0124%, sulfur 0.0282%, molybdenum 0.0125%, and nickel 0.0015%.

### Electrochemical test

Electrochemical studies: A PalmSens3 potentiostat was used along with a three-electrode cell at 298K. A graphite rod (*d* = 3 mm, *l* = 4 cm) served as an auxiliary electrode and the saturated calomel electrode (SCE) as a reference electrode.

The potentiodynamic polarization curves were obtained by scanning the electrode potential at least 250 mV vs. E_OCP_ with a sweep rate of 1 mVs^−1^. The measurements were conducted under atmospheric conditions. To check the reproducibility, every experiment was repeated three times.

### Weight loss measurements

The weight loss tests (repeated three times) were performed at 298K using 100 ml of the aerated corrosion solution (0.1 M H_2_SO_4_) in the absence or presence of the studied inhibitors (Obi-Egbedi et al., 2011a). Prior to immersion of mild steel coupons [Size (W × L × D): 12.7 mm × 50.8 mm × 6 mm], the mirror like coupons (abraded with emery papers of various grade sizes: 400, 600, 1,000, and 1,500) were rinsed with double distilled water, cleaned in a sonicating acetone bath for 15 min, followed by sonication in ethanol. The weight difference (weighed using Scaltec Analytical Balance model SBC 31) between the mild steel coupons weight at 6 h time and the initial weight of the coupons was taken as the weight loss which was used to calculate the corrosion rate given by:

(1) $$\begin{array}{r} \rho =\frac{\Delta W}{\Delta t} \end{array}$$

where:

ρ is the corrosion rate, Δ*w* is the weight loss, A is the area of the coupon and *t* is the corrosion time.

### Computational details

#### Molecular dynamics simulation

Adsorption Locator module in Materials studio 7.0 has been used to build 4MT, 2MA molecules, Fe(1 1 1) (Khaled et al., 2012) and Fe_2_O_3_ (1 1 1) (Bowker et al., 2012) surface (Akkermans et al., 2013). The molecular dynamic simulations of the interaction between the studied inhibitors and the two surfaces were carried out in the simulation box [Fe(111)–16.21Å × 16.21Å×7.46Å; Fe_2_O_3_(111)–14.39Å × 13.46Å × 5.88Å) using periodic boundary conditions with a of 20 Å vacuum along the C-axis. The solvent (water) effect was simulated by loading 50 water molecules (geometrically optimized using COMPASS forcefield) onto the simulation box together with the studied molecules (using the same optimization algorithm). For the charged structures (protonated forms of triazole and pyridine rings; Supplementary Figure 1), a positive charge was applied on the protonated nitrogens in both of the structures (using QEq method).

The Metropolis Monte Carlo method was used to determine the adsorption configurations (COMPASS, force field) of the interaction between the 4MT, 2MA, and the Fe (1 1 1) or Fe_2_O_3_ (1 1 1) surface (Khaled et al., 2012; Yesudass et al., 2016).

#### Quantum chemical calculations

The quantum chemical calculations (Obi-Egbedi et al., 2011b; Obot et al., 2015) were performed by using DFT: B3LYP/6-31G^*^ as implemented in the Spartan 16 software (Wavefunction Inc., Irvine, CA). All energy minima were characterized by performing a vibrational analysis to ensure the lack of imaginary frequencies (Berisha et al., 2017). The DFT: B3LYP/6-31G^*^ permits calculating, in a reproducible manner, the molecular parameters (electronegativity, global hardness, and softness, electron affinity and ionization potential, etc.; Geerlings et al., 2003; Gece, 2008; Berisha et al., 2015b; Obot et al., 2015).

DFT molecular parameters, their meaning and the equations used for their calculations are presented in the Table 1.

**Table 1:** Molecular DFT parameters definitions.

**Formula**	**Meaning**
*X≈-1/2(E_*HOMO*_+E_*LUMO*_)* (2)	Electronegativity (χ) is the quantity of the influence of an electron or a group of atoms to attract electrons toward them.
*η≈-1/2(E_*HOMO*_-E_*LUMO*_)* (3)	Chemical hardness (η) is the amount of the opposition of an atom to a charge transfer.
*σ≈1/η = -2(E_*HOMO*_-E_*LUMO*_)* (4)	Chemical softness (σ) is the measure of the ability of an atom or a group of atoms to accept electrons.
*IP≈-E_*HOMO*_*(5)	Ionization potential (IP) represent the quantity of energy that is for the removal of an electron from a molecule.
*EA≈-E_*LUMO*_*(6)	Electron affinity (EA) is defined as the energy released when a proton is added to a system.
ω = μ^2^/2η (7)	Electrophilicity index (ω) is a measure of energy lowering due to maximal electron flow between donor and acceptor Parr et al., 1999.

#### DFT calculations with a plane wave basis set

Geometry optimization with CASTEP (Clark et al., 2005) is done through generalized gradient approximations (GGE) using PBE functional (Hammer et al., 1999). Self-consistent iteration method (SCF; Payne et al., 1992) was used for geometry optimization (1,000 iteration steps, using energy convergence of 2.0e-5 eV/atom). A Fe cluster of 3.25 Å (in a vacuum box of 10 Å^3^) and Fe_2_O_3_ cluster of 3.5 Å in diameter (in a vacuum box of 15 Å^3^) were used for the calculations. The same energy optimization parameters and vacuum box sizes were used also to compute energies of the adsorbate molecules and the DOS (Density of States) plots.

The adsorption energy (E_ads._) (Gao et al., 2015) was calculated as:

(2) $$\begin{array}{r} E\left(ads\right)=-\left(Etotal+Ecluster-Eadsorbate\right) \end{array}$$

where:

E_total_ is the energy of the Fe or Fe_2_O_3_ cluster/ adsorbate, E_adsorbate_ is the energy of the isolated adsorbate molecules (2MA or 4MA) and E_cluster_ is the energy of the isolated, fully relaxed Fe or Fe_2_O_3_ clusters.

## Results and discussion

**Figure 1:**
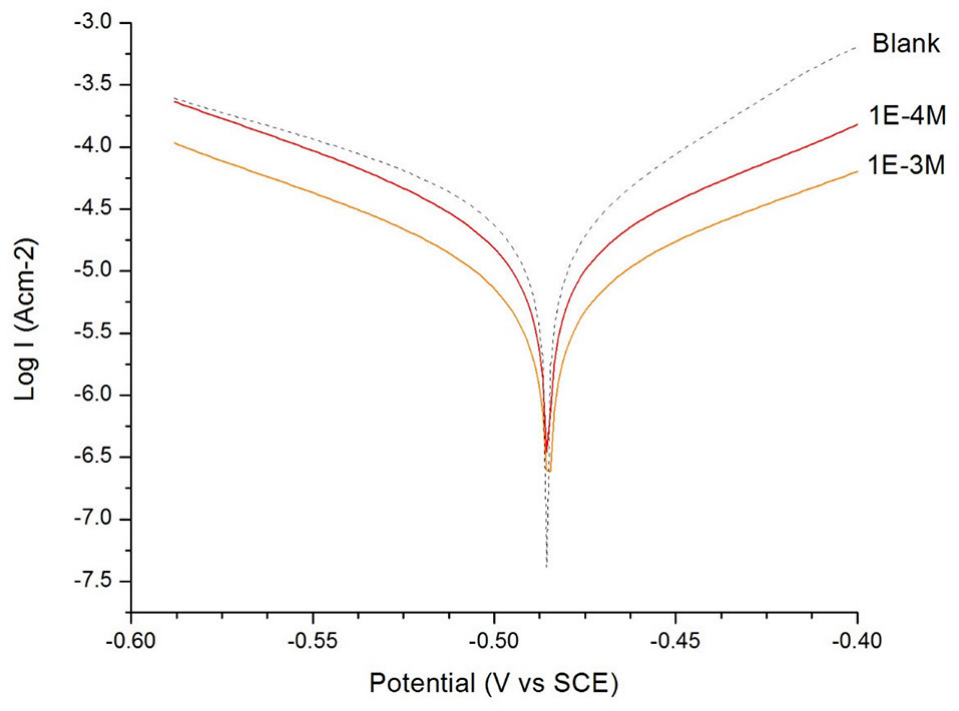
Tafel plot of the mild steel electrode measured in H_2_SO_4_ solution (*c* = 0.1 M): in the absence and in the presence of 1 × 10^−3^ M and 1 × 10^−4^ M of 2-mercaptonicotinic acid (2MA).

**Figure 2:**
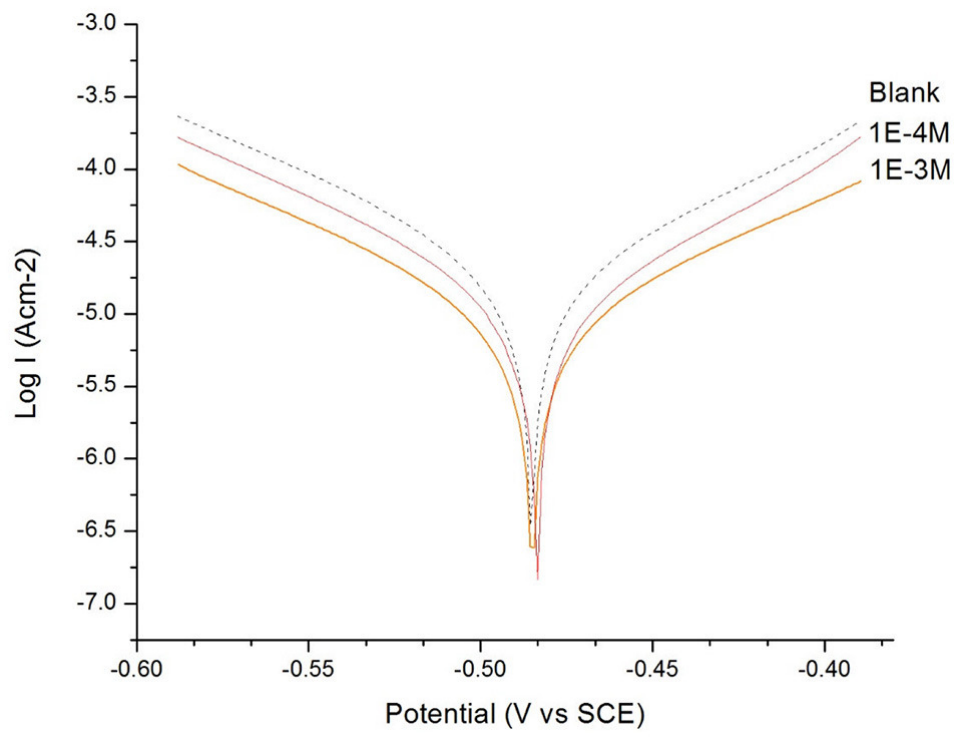
The Tafel plot of the mild steel electrode measured in H_2_SO_4_ solution (*c* = 0.1 M): in the absence and in the presence of 1 × 10^−3^ M and 1 × 10^−4^ M of 4-methyl-4H-1,2,4-triazole-3-thiol (4MT).

In Figures 1, 2, the anodic and cathodic polarization curves of a mild steel electrode in H_2_SO_4_ solution (*c* = 0.1 M) are presented in the absence and presence of 1 × 10^−3^ M, 1 × 10^−4^ M of 2MA and 4MT, respectively, at 298 K. The IE (%) was calculated using the following Equation (9):

(3) $$\begin{array}{r} IE\left(\%\right)=\frac{\left[i_{absenceof\;inhibitor}^{corr.}-i_{presenceof\;inhibitor}^{corr.}\right]}{\left[i_{absenceof\;inhibitor}^{corr.}\right]}100 \end{array}$$

The electrochemical parameters: corrosion potential (E_corr_) and corrosion current density (i_corr_), were determined from the intersection of anodic and cathodic Tafel slopes and are presented in Table 2.

**Table 2:** The inhibition efficiency and other electrochemical parameters of the 2MA and 4MT inhibitors at 1 × 10^−3^ M and 1 × 10^−4^ M toward the mild steel in 0.1 M H_2_SO_4_corrosion media.

**Parameters**	**Fe**	**1 × 10^−4^ M 4MT**	**1 × 10^−3^ M 4MT**	**1 × 10^−4^ M 2MA**	**1 × 10^−3^ M 2MA**
bc (V/dec)	0.058	0.092	0.089	0.090	0.097
ba (V/dec)	0.103	0.082	0.090	0.074	0.115
Ecorr (V)	−0.470	−0.484	−0.478	−0.475	−0.477
Rp (Ω)	639.4	2471.7	2579.8	1787.4	2467.6
IE (%)	−	63.13	70.13	61.03	63.17
± *SD*		1.8	1.3	1.5	1.4

From the Tafel plot in Figures 1, 2, it is evident that the adsorption of the 2MA and 4MT, respectively, molecules onto the mild steel surface strongly lowers the corrosion current of the mild steel in this aggressive media, reflecting a high value of the corrosion inhibition efficiency up to 70.13 ± 1.3% for the 2MA molecule and 63.17 ± 1.4% 4MT. This is in good agreement with the corrosion inhibition efficiency measured through the weight loss measurements (69.85 ± 2.2% for 2MA and 64.52 ± 2.6% for 4MT).

The Resistance of the polarization (Rp) from Tafel extrapolation method was calculated by employing the Stern–Geary Equation (Larabi et al., 2004) Equation (10):

(4) $$\begin{array}{r} R_{p}=\frac{\beta_{a}\beta_{c}}{2.303\left(\beta_{a}\beta_{c}\right)}\frac{1}{i_{corr.}} \end{array}$$

Table 2 shows that upon increasing the inhibitor concentration, a larger polarization resistance is obtained with both compounds, this reflects the adsorption of the inhibitor on the metal surface which passivates efficiently the active sites and inhibits the corrosion (Mansfeld, 1981; Mohsenifar et al., 2016).

The values of cathodic and anodic Tafel slopes (bc, ba) change when 2MA or 4MT are present in the solution. The Tafel slope differences imply that both of molecules affect the kinetics of the hydrogen evolution reaction (Quartarone et al., 2006). This corresponds to a higher energy barrier for proton discharge, resulting in less gas evolution (Selvi et al., 2003). The studied molecules do not change significantly the corrosion potential, indicating a mixed inhibitor (Berisha et al., 2015b).

**Figure 3:**
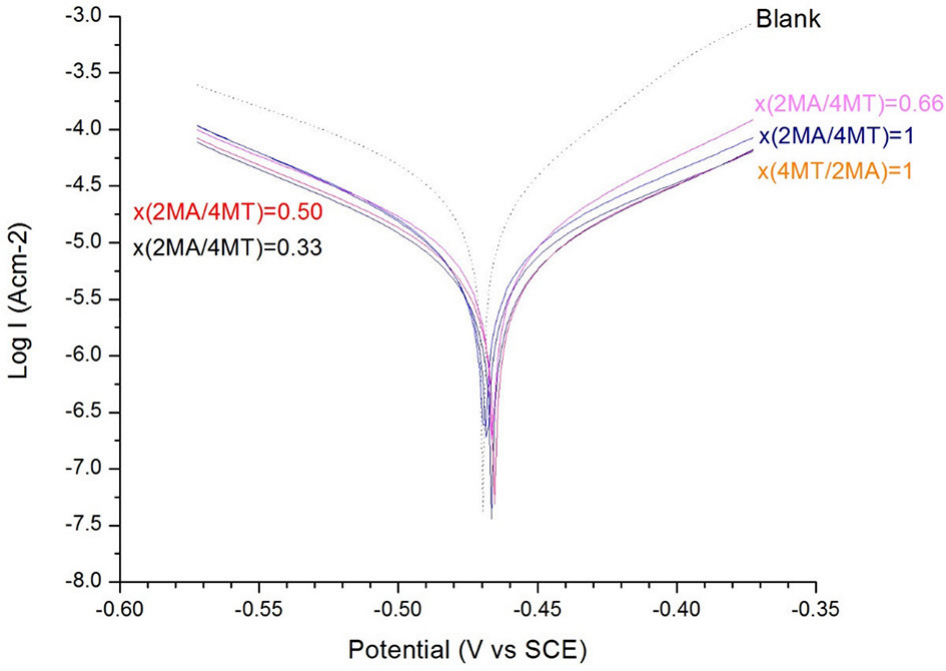
The Tafel plot of the mild steel electrode measured in H_2_SO_4_ (*c* = 0.1 M) solution: with and without different molar ratio of x(2MA/(4MT) = 1, 0.66, 0.5, 0.33, and x(4MT/(2MA) = 1 (*c* = 1 × 10^−3^ M for both of the molecules).

From the data presented in Table 3 (calculated from Figure 3) it is obvious that the use of the mixed inhibitors, in fact, does not have an important impact on the IE.

**Table 3:** The inhibition efficiency of the mixed inhibitors (2MA/4MT) at different molar ratios from 0 to 1 (each 1 × 10^−3^ M) toward the mild steel in 0.1 M H_2_SO_4_ corrosion media.

**Parameters**	**Fe**	**x(2MA/4MT)**				
		**1**	**0.66**	**0.50**	**0.33**	**x(4MT/2MA) = 1**
bc (V/dec)	0.058	0.089	0.098	0.109	0.109	0.097
ba (V/dec)	0.103	0.090	0.082	0.103	0.105	0.115
Ecorr (V)	−0.470	−0.478	−0.468	−0.470	−0.471	−0.477
Rp (Ω)	639.4	2579.8	2284.8	2977.1	2801.3	2467.6
IE (%)	−	70.13	66.20	69.44	67.18	63.17
± *SD*		1.3	1.1	1.3	1.5	1.2

### Monte carlo results

**Figure 4:**
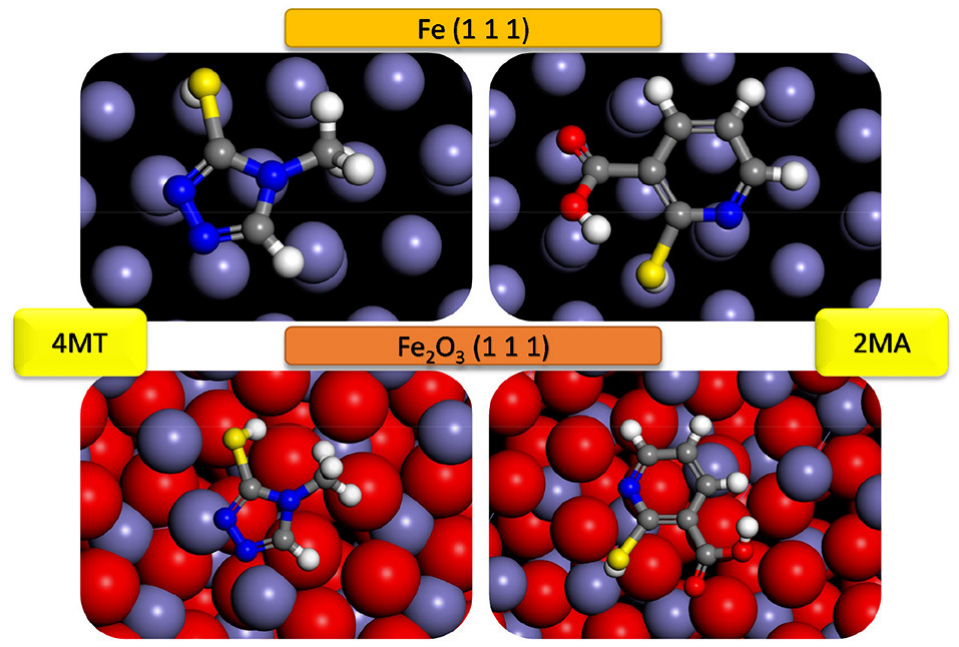
Most likely configuration for the adsorption of 4MT and 2MA on Fe (111) and Fe_2_O_3_ (111) substrate attained by the adsorption locator module.

The most stable adsorption configuration of 2MA and 4MT molecules onto Fe (111) and Fe_2_O_3_ (111) obtained by simulated annealing using the Adsorption Locator module are presented in Figure 4. Both of the studied molecules prefer planar adsorption onto the studied surfaces in each case with the hydrogen atom of the thiol group pointing toward the surface (in the case of 4MT and 2 MA) and the hydrogen atom (2MA) pointing out of the surface.

The results of the Monte Carlo simulation are presented in Tables 4, 5. In this study, the substrate energy (iron surface) is taken as zero. In addition, adsorption energy in kcal/mol, represents the energy released upon the adsorption of the relaxed adsorbate molecules onto the substrate.

**Table 4:** The results calculated by the Monte Carlo simulation of 4MT and 2MA conformations on iron (111) surface.

**Structures**	**Total energy**	**Adsorption energy**	**Rigid adsorption energy**	**Deformation energy**	**dEad/dNi**
**4-METHYL-4H-1,2,4-TRIAZOLE-3-THIOL (4MT)**					
4MT	−2.764				
Fe (1 1 1) - 1	−68.015	−65.251	−54.465	−10.786	−65.251
Fe (1 1 1) - 2	−64.870	−62.107	−51.404	−10.702	−62.107
Fe (1 1 1) - 3	−64.201	−61.437	−50.691	−10.746	−61.437
Fe (1 1 1) - 4	−63.656	−60.893	−50.413	−10.479	−60.893
Fe (1 1 1) - 5	−62.381	−59.617	−49.029	−10.588	−59.617
Fe (1 1 1) - 6	−61.875	−59.112	−49.533	−9.579	−59.112
Fe (1 1 1) - 7	−61.356	−58.592	−47.930	−10.663	−58.592
Fe (1 1 1) - 8	−61.113	−58.349	−49.116	−9.233	−58.349
Fe (1 1 1) - 9	−60.908	−58.145	−48.205	−9.940	−58.145
**2-MERCAPTONICOTINIC ACID (2MA)**					
2MT	−17.678				
Fe (1 1 1) - 1	−98.922	−81.244	−65.368	−15.876	−81.244
Fe (1 1 1) - 2	−96.189	−78.512	−62.355	−16.156	−78.512
Fe (1 1 1) - 3	−94.375	−76.698	−63.699	−12.999	−76.698
Fe (1 1 1) - 4	−93.870	−76.192	−62.734	−13.458	−76.192
Fe (1 1 1) - 5	−93.077	−75.399	−60.003	−15.396	−75.399
Fe (1 1 1) - 6	−92.281	−74.603	−61.529	−13.075	−74.603
Fe (1 1 1) - 7	−91.590	−73.912	−60.066	−13.846	−73.912
Fe (1 1 1) - 8	−91.218	−73.540	−61.521	−12.019	−73.540
Fe (1 1 1) - 9	−91.006	−73.329	−59.263	−14.065	−73.329
Fe (1 1 1) - 10	−90.229	−72.551	−60.361	−12.190	−72.551

**Table 5:** The results calculated by the Monte Carlo simulation of 4MT and 2MA conformations on iron oxide (111) surface.

**Structures**	**Total energy**	**Adsorption energy**	**Rigid adsorption energy**	**Deformation energy**	**dEad/dNi**
**4-METHYL-4H-1,2,4-TRIAZOLE-3-THIOL (4MT)**					
4MT	−2.764				
Fe_2_O_3_ (1 1 1) - 1	−64.138	−61.374	−53.267	−8.107	−61.374
Fe_2_O_3_ (1 1 1) - 2	−62.493	−59.729	−52.407	−7.323	−59.729
Fe_2_O_3_ (1 1 1) - 3	−62.000	−59.237	−48.982	−10.255	−59.237
Fe_2_O_3_ (1 1 1) - 4	−61.303	−58.540	−48.432	−10.107	−58.540
Fe_2_O_3_ (1 1 1) - 5	−60.768	−58.004	−49.698	−8.306	−58.004
Fe_2_O_3_ (1 1 1) - 6	−60.147	−57.383	−48.813	−8.570	−57.383
Fe_2_O_3_ (1 1 1) - 7	−55.071	−52.308	−42.532	−9.776	−52.308
Fe_2_O_3_ (1 1 1) - 8	−52.693	−49.929	−39.726	−10.203	−49.929
Fe_2_O_3_ (1 1 1) - 9	−52.342	−49.578	−39.679	−9.899	−49.578
**2-MERCAPTONICOTINIC ACID (2MA)**					
2MT	−17.678				
Fe_2_O_3_ (1 1 1) - 1	−109.465	−91.787	−83.474	−8.313	−91.787
Fe_2_O_3_ (1 1 1) - 2	−107.366	−89.688	−81.098	−8.590	−89.688
Fe_2_O_3_ (1 1 1) - 3	−105.785	−88.107	−77.100	−11.007	−88.107
Fe_2_O_3_ (1 1 1) - 4	−105.019	−87.342	−78.622	−8.720	−87.342
Fe_2_O_3_ (1 1 1) - 5	−104.384	−86.706	−77.317	−9.389	−86.706
Fe_2_O_3_ (1 1 1) - 6	−104.000	−86.323	−77.291	−9.032	−86.323
Fe_2_O_3_ (1 1 1) - 7	−103.459	−85.781	−77.881	−7.900	−85.781
Fe_2_O_3_ (1 1 1) - 8	−102.989	−85.312	−76.154	−9.158	−85.312
Fe_2_O_3_ (1 1 1) - 9	−100.297	−82.619	−76.116	−6.504	−82.619
Fe_2_O_3_ (1 1 1) - 1	−98.032	−80.355	−71.490	−8.864	−80.355

The adsorption energy is defined as the sum of the rigid adsorption energy and the deformation energy for the adsorbate components. The rigid adsorption energy reports the energy, in kcal/mol, released (or required) when the unrelaxed adsorbate components (i.e., before the geometry optimization step) are adsorbed on the substrate.

**Figure 6** presents the adsorption energy distribution of the 4MT and 2MA molecules on Fe (1 1 1), respectively, Fe_2_O_3_ (1 1 1). The adsorption energy of 4MT is −68.01 kcal/mol onto Fe (1 1 1) and −64.38 kcal/mol onto Fe_2_O_3_ (1 1 1) surface, whereas the adsorption energy for 2MA reaches −98.92 kcal/mol in the case Fe (1 1 1) and −109.46 kcal/mol for the Fe_2_O_3_ (1 1 1) surface. These relatively large values for both molecules reflect their strong adsorption on the studied surfaces. The adsorption energies through Monte Carlo simulations (Table 6) were calculated also with neutral and protonated 4MT and 2MA molecules in the absence and presence of water (the adsorption energy distribution of the adsorbate and the appropriate configuration are found in the Supplementary Figures 2–5).

**Table 6:** Adsorption energies calculated by the Monte Carlo simulation of 4MT and 2MA (in neutral and protonated form) on Fe (1 1 1) and Fe_2_O_3_ (1 1 1) surface, in the presence and absence of water.

**System**	**Eads. (kcal/mol)**
Fe/4MT (vacuum)	−68.01
Fe/2MA (vacuum)	−98.92
Fe_2_O_3_/4MT (vacuum)	−64.38
Fe_2_O_3_/2MA (vacuum)	−109.46
Fe/4MT (water)	−77.59
Fe/2MA (water)	−106.80
Fe_2_O_3_/4MT (water)	−63.25
Fe_2_O_3_/2MA (water)	−94.25
Fe/4MT-H^+^ (vacuum)	−143.05
Fe / 2MA-H^+^ (vacuum)	−233.55
Fe_2_O_3_/ 4MT-H^+^ (vacuum)	−136.75
Fe_2_O_3_/2MA-H^+^ (vacuum)	−240.55
Fe/4MT-H^+^ (water)	−159.15
Fe / 2MA-H^+^ (water)	−254.99
Fe_2_O_3_/ 4MT-H^+^ (water)	−138.75
Fe_2_O_3_/2MA-H^+^ (water)	−243.25

The adsorption geometries for 2MA and 4MT are in all cases in the planar configurations. From the calculated results, it is evident that the most important energies, are obtained for the protonated forms of 2MA and 4MT in the presence of water. In this case for the Fe (111) surface the adsorption energy of 2MA-H^+^ has a relatively large value −254.99 kcal/mol, to be compared with that 4MT-H^+^ −159.15 kcal/mol. These large values indicate a strong interaction of these molecules with the studied surfaces.

### Quantum chemical calculations

**Figure 5:**
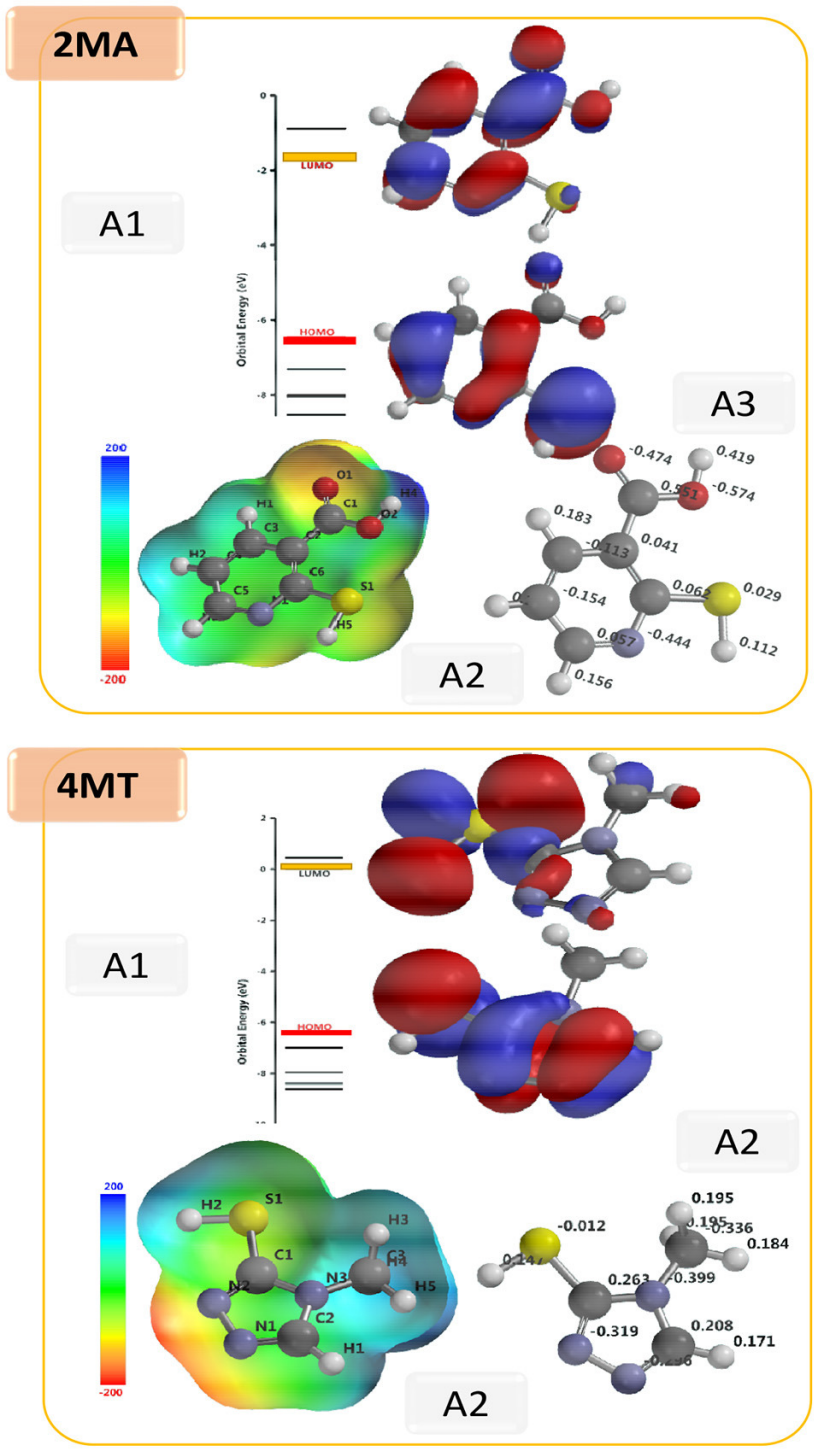
Optimized structures: A1; HOMO and LUMO; A2, Electrostatic potential map and A3, Mulliken charge for 2MA and 4MT molecules. (B3LYP/6-31G^*^ results *in vacuo*).

The shape and symmetry of the HOMO and the LUMO are the central factors for the estimation of the reactivity of a compound (Gece, 2008; Obot et al., 2015). The analysis of the HOMO highlights the areas of the molecule that can donate electrons to electrophilic species while the analysis of the LUMO predicts the regions of the molecule with high affinity to accept electrons from nucleophilic species. In 2MA (Figure 5), the HOMO orbital has the highest electron density on the N1-C6, C4-C5 single bonds, and C2 = C6 double bond, this points out that these are the areas of the molecule with the highest affinity to donate electrons; the LUMO is localized on C3, C5, and C6 atoms. In the 4MT molecule, the HOMO orbital is extended over a large region between the C2-N3, N1-N2 single bonds, C2 = N1 double bond and S atom; the LUMO is strongly localized at the −SH group. Ionization potential is directly related to the energy of HOMO orbital and the electron affinity to that of LUMO orbital. Our results show that the HOMO and the LUMO are delocalized throughout molecule. In the investigated compounds (Table 7), the E_HOMO_ is larger in 4MT, consequently, 4MT would have the highest affinity to adsorb onto the metal surface and to offer electrons (the lone pair electrons on S and N atoms) to the unoccupied iron d-orbital (Fouda and Ellithy, 2009).

**Table 7:** The molecular properties of the investigated compounds using: DFT, B3LYP/6-31G (d,p). Results in *vacuo*.

**Molecule**	**4MT**	**2MA**
E_(*HOMO*)_	−6.470	−6.370
E_(*LUMO*)_	−1.720	0.020
ΔE	−4.750	−6.390
μ	1.060	4.920
IP	6.470	6.370
EA	1.720	−0.020
χ	4.095	3.175
η	2.375	3.195
σ	0.421	0.313
ω	0.237	3.788

*All energies are in eV*.

These results are in good agreement with experimentally observed inhibition efficiencies. A negative value for E_HOMO_ suggests a physical adsorption of the organic molecule (Yurt et al., 2006). The energy of the LUMO shows the tendency of the molecule to accept electrons from an electron-rich species. The molecule with the lowest energy of the LUMO has the highest tendency to accept electrons. This is in perfect agreement with the calculated values E_LUMO_ 4MT < E_LUMO_ 2MA implying that 4MT would preferentially accept electrons from the metal surface more than 2MA.

The energy difference between the E_HOMO_ and the E_LUMO_, ΔE, expresses the reactivity of the molecule by comparison with other compounds. The molecules with the lowest ΔE value express the highest affinity reactivity and would favorably interact with the surface, this is not in agreement with what we found ΔE (4MT) > ΔE (2MA).

The global hardness and softness parameters are commonly described in terms of hard-soft-acid-base (HSAB) theory; by this theory, soft acids act preferentially with soft bases and hard ones with hard bases. Metals are normally considered to be soft acids (Obi-Egbedi et al., 2011b); therefore, they would favorably interact with the inhibitors that display low η and high σ values.

The σ value for the investigated compounds is higher for 4MT > 2MA which fully agrees with the experimentally determined inhibition efficiency of the inhibitors. The dipole moment tells about the polarity of the molecule. It has been established that an increased inhibition efficiency is related to an increased dipole moment (Obi-Egbedi et al., 2011b; Berisha et al., 2015b). The dipole moment for the studied compounds is the higher for 4MT, which fits with our experimental results. Certain molecular properties do not only specify the reactivity of molecules but they also designate the position selectivity in the molecules, i.e., the areas on which particular types of reactions are more likely to take place. One of the parameters in this group is the partial atomic charge on the atoms. Frequently the interaction between the metal and the inhibitor is occurs favorably on the atom bearing the utmost negative charge. The Mulliken atomic charges for 4MT and 2MA are shown in Figure 5 and the highest values of the negative charge on the molecules are to be found on the heteroatoms, suggesting that these centers have the maximum electron density and would preferentially interact with the metal surface. In order to obtain more realistic results, DFT calculations were also performed using water as a solvent with 2MA and 4MT in neutral and protonated state. The results are very similar and lead to the same conclusions as above (Supplementary Table 1) indicating that solvation of the surface and molecules is not determining in the corrosion process.

### DFT calculations with a plane wave basis set

As Monte Carlo calculations are based on the use of the force fields and as there is no electron correlation (Kalos and Whitlock, 2008; Akkermans et al., 2013; Schattke and Muiño, 2013), more accurate calculations are used to estimate a correct adsorption energy and geometries. This is performed through DFT periodic calculations with a plane wave basis set.

**Figure 6:**
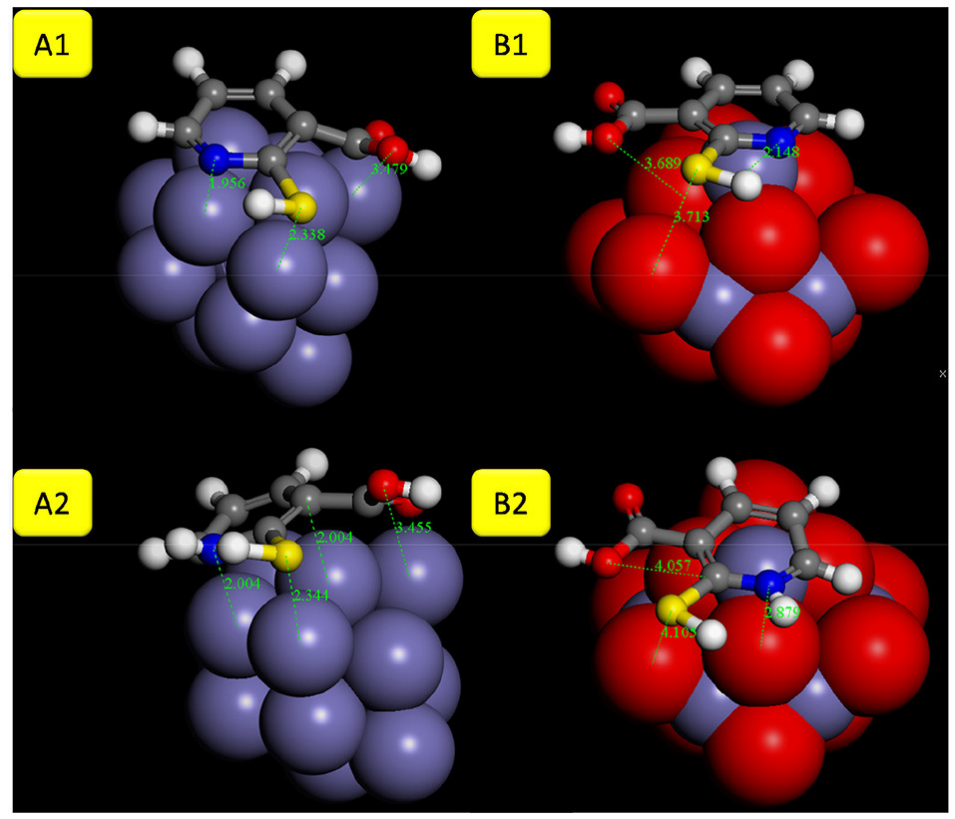
The optimized geometries with adsorption distances of 2MA molecule (**A1/B1** neutral and **A2/B2** protonated form) onto: **(A)** Fe and **(B)** Fe_2_O_3_ cluster.

The planar geometry is preferred for the adsorption of the 2MA molecule onto the Fe surface (Figure 6). The S atom of the thiol group of the 2MA molecule is positioned at 2.33 Å from the Fe nanocluster, whereas the N of the pyridine ring is at 1.95 Å. The hydrogen atom of the thiol group is oriented away from the surface, indicating that the adsorption is taking place through the S atom, pyridine ring and the carboxyl group (positioned almost flat onto the cluster surface).

**Figure 7:**
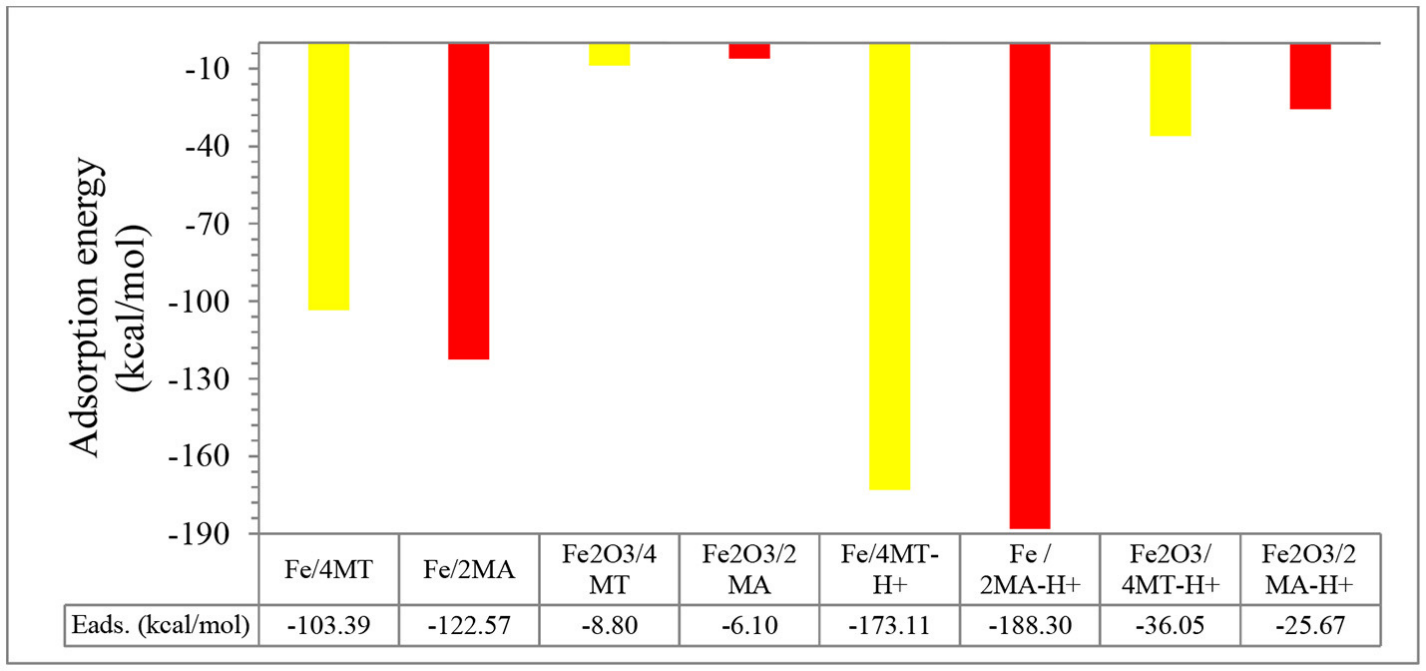
Adsorption energies for 2MA and 4MT (in neutral and protonated forms) onto Fe and Fe_2_O_3_ clusters.

**Figure 8:**
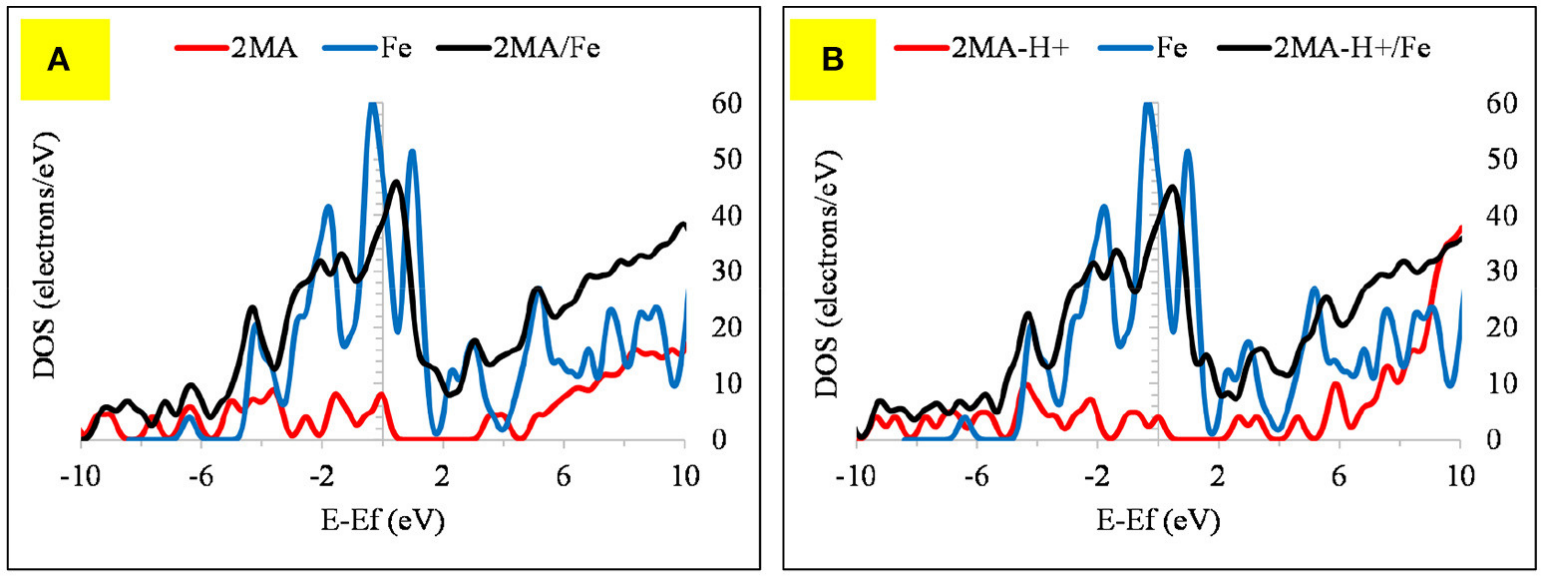
The DOS plot for: **(A)** 2MA, Fe and 2MA/Fe and **(B)**. **(A)** 2MA-H+, Fe and 2MA-H+/Fe cluster system.

The computed (by periodic DFT calculations) adsorption energy of −122.57 kcal/mole (Figure 7) supports this strong interaction of this molecule with the iron cluster surface, this also evidenced by analyzing the DOS plot (Figure 8A).

The Fermi level of the Fe cluster after the interaction with the 2MA molecule shifts upwards, with a peak broadening due to the overlap of the valence band orbitals of the Fe cluster with those of the 2MA molecule (Cho et al., 2008; Raouafi et al., 2016).

In the protonated form, the distances of the 2MA molecules onto the cluster are slightly increased, the S atom is positioned at 2.34 Å, the N of the pyridine ring 2.0 Å and the adsorption energy in this case is increased up to −188.30 kcal/mol. The pyridine ring interacts with iron surface through the nitrogen atom via coordinative Fe-**N** bonding (Gu et al., 2001). This interaction is evidenced in the DOS plot (Figure 8B) by a more pronounced peak broadening compared with the neutral form.

**Figure 9:**
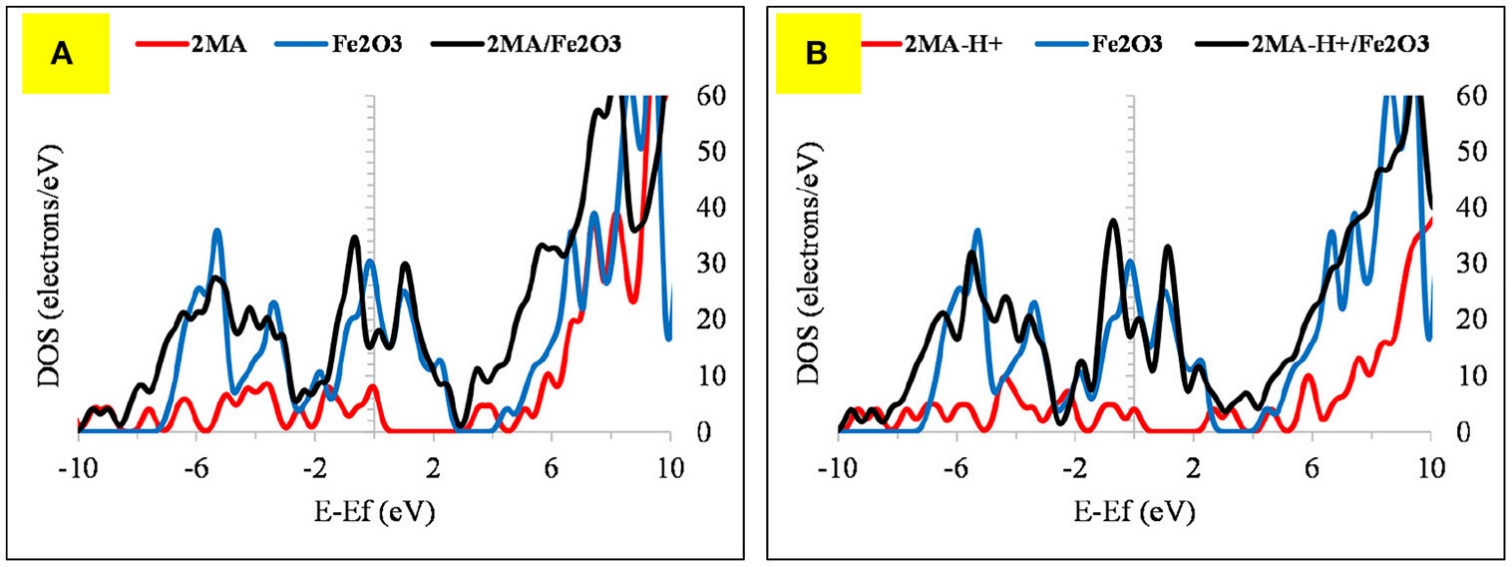
The DOS plot for: **(A)** 2MA, Fe_2_O_3_ and 2MA/Fe_2_O_3_ and **(B) (A)** 2MA-H+, Fe_2_O_3_ and 2MA-H+/ Fe_2_O_3_ cluster system.

The adsorption of 2MA onto the Fe_2_O_3_ surface is planar as with the 4MA. The S atom distance from the surface of this cluster is at 3.71 Å, whereas the N of the pyridine ring is at 2.14 Å. These distances are larger than in the case of Fe (1.375 Å for S atom and 0.162 Å for pyridine ring nitrogen). The hydrogen atom of the thiol group is parallel to the surface, as well as the pyridine ring and the carboxyl group. The relatively insignificant value of adsorption energy −6.1 kcal/mole (2MA) and −25.6 kcal/mol (2MA-H^+^) points out a weaker interaction at this surface as proved also via evaluating the DOS plot (Guo et al., 2017b) and the increase of the adsorption distance of the molecule compared to the Fe. The upward shift of the Fermi level after the interaction with the 2MA or 2MA-H^+^ is less apparent (Figures 9A,B). Minor peak broadening occurs as a result of the slight overlap of the valence band orbitals of the Fe_2_O_3_ cluster with the valence orbitals of 2MA molecule (Jarvis et al., 2015).

**Figure 10:**
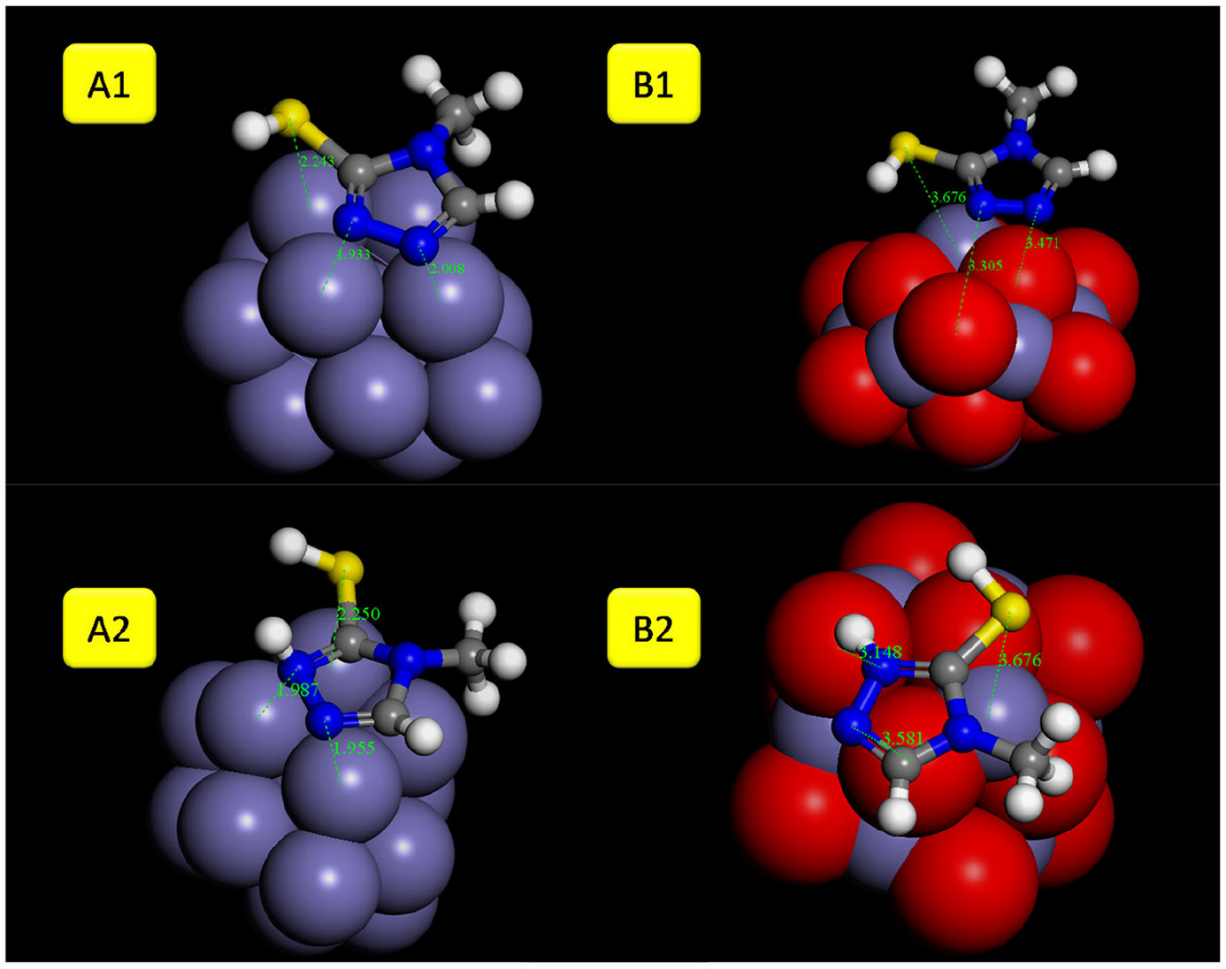
The optimized geometries with adsorption distances of 4MT molecule (**A1/B1** neutral and **A2/B2** protonated form) onto: **(A)** Fe and **(B)** Fe_2_O_3_ cluster.

**Figure 11:**
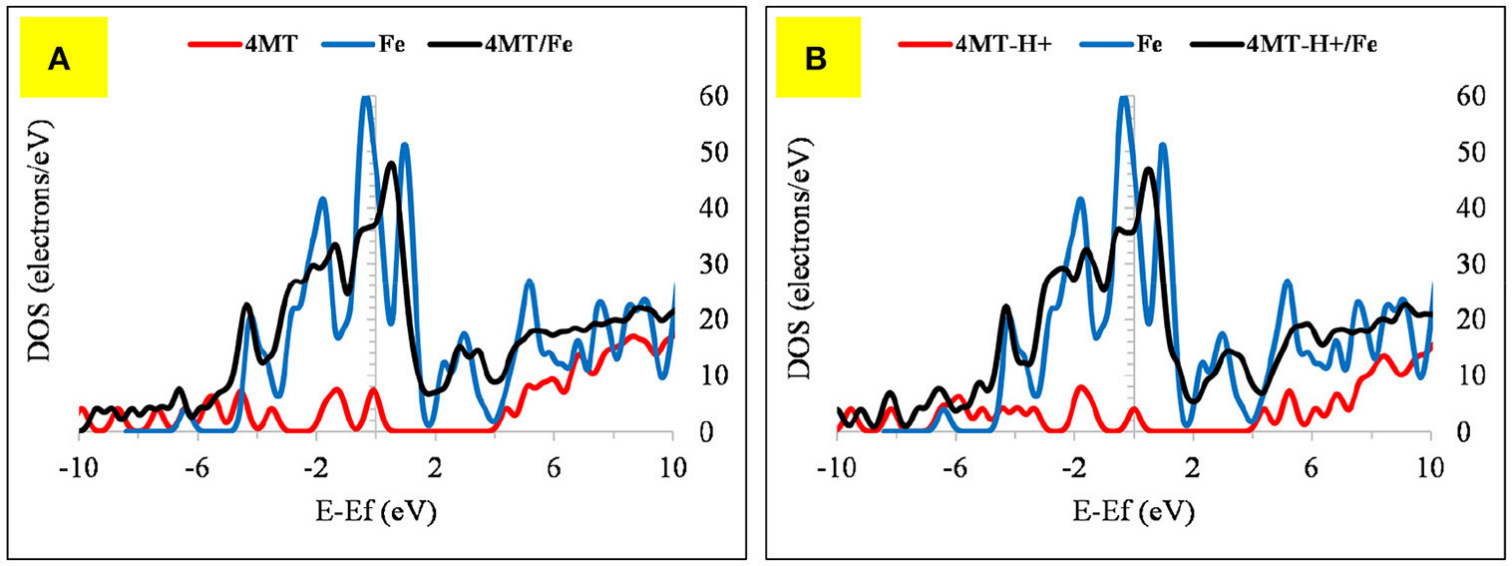
The DOS plot for: **(A)** 4MT, Fe and 4MT/Fe and **(B) (A)** 4MT -H+, Fe and 4MT-H+/Fe cluster system.

**Figure 12:**
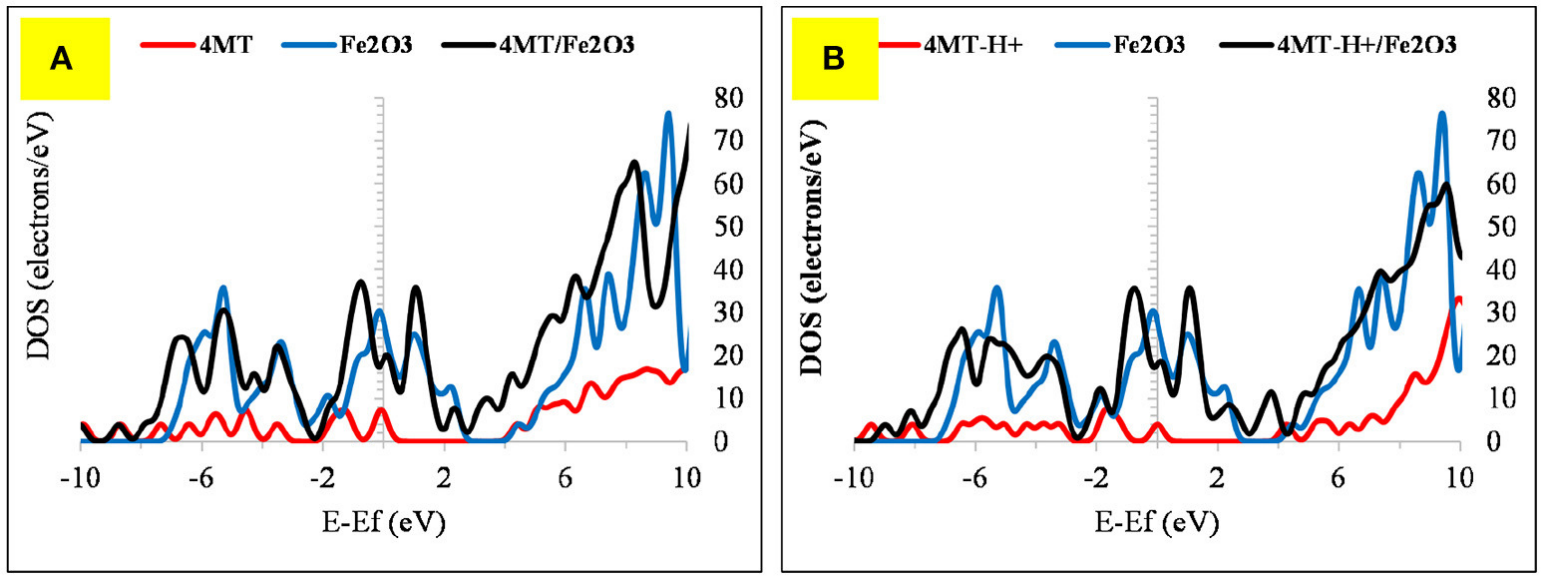
The DOS plot for: **(A)** 4MT, Fe_2_O_3_ and 4MT/ Fe_2_O_3_ and **(B) (A)** 4MT -H+, Fe_2_O_3_ and 4MT-H+/ Fe_2_O_3_ cluster system.

The 4MT molecule adsorbs flat onto the Fe surface (Figure 10). The S atom distance of the thiol group from the Fe surface is 2.43 Å, the N of the triazole ring next to the thiol group is at 1.99 Å. Contrary to the 2MA adsorption, the hydrogen atom of the thiol group is facing the surface, indicating that the adsorption is mainly due to the nitrogen atoms of the azole ring. The calculated adsorption energy is lower by about −19.18 kcal/mole compared to the 2MA. This surface/adsorbate interaction is supported by analyzing the upward shift of the Fermi level (with peak broadening) of the 4MT or 4MT-H+ /Fe surface (Figures 11, 12) (Jarvis et al., 2015; Raouafi et al., 2016).

In the protonated form, the distances of the 4MT molecule onto the cluster are slightly decreased, the S atom is positioned at 2.25 Å, the N of the ring is at 1.98 Å. The hydrogen atom of the thiol group is facing out the surface, exposing the S atom to the surface.

The flat geometry is the favored one for both 4MT and 4MT-H^+^ (Figure 10) onto the Fe_2_O_3_ cluster. The S atom of the thiol group of the 4MT molecule is at 3.47 Å (for 4MT), respectively, for 3.67 Å from the clusters surface. The N atom (next to the thiol group) of the triazole ring is at 3.30 Å for 4MT and 3.14 Å for 4MT-H^+^. The adsorption energies are greater for the protonated than the neutral form of 4MT.

### The inhibition mechanism

In general the corrosion inhibition mechanism in acid medium is the adsorption of inhibitor molecule onto the metals surface by one of the four types of adsorption: (1) electrostatic attraction between charged molecules and the charged metal, (2) interaction of unshared electron pairs in the molecule with the metal, (3) interaction of π-electrons with the metal, and (4) a combination of the above (Bhajiwala and Vashi, 2001; Singh et al., 2011). For the physical adsorption to take place in acidic medium, the presence of both a metal surface (with vacant low-energy electron orbitals) and charged species in the solution (a molecule having reasonably loosely bound electrons or heteroatom with lone pair electrons) are necessary.

Coordinate covalent bond formation between electron pairs of unprotonated S atom and N atoms of aromatic rings with metal surface can take place for both of the molecules during their adsorption (Gu et al., 2001). This leads to a protective film on mild steel surface that reduces the corrosion rate in good agreement with the experimental results. Through the above calculation we show that the main interactions take place through the heteroatoms. They show that that these molecules lie flat on the surface (Guo et al., 2017a). Finally an organic film forms on the surface with a interaction energy in the range of −122.57 kcal/mol for 2MA-H^+^, respectively, −103.39 for 4MT-H^+^ that prevents the corroding species from reaching the metallic surface (Singh and Quraishi, 2010; Karthik and Sundaravadivelu, 2013).

## Conclusion

The inhibition efficiency of 4MT and 2MA has been explored experimentally and by quantum calculations. Potentiodynamic measurements reveals a strong inhibition behavior of these molecules toward mild steel corrosion. Different calculations were performed to rationalize these results: theoretical parameters [E_HOMO_, E_LUMO_, ΔE (HOMO-LUMO), global hardness (η), global softness (σ), and the dipole moment (μ)], Monte Carlo simulations and DFT quantum chemical calculations using periodic systems.

The calculations permit to shed some light on the fundamental reasons for this reduced corrosion rates. The active centers which would interact with the metal surface are determined as well as the adsorption geometries and energies. Experimental and theoretical results show that the inhibition efficiency is higher for 2MA due to the presence of the pyridine nitrogen, carboxyl, and the sulfur atom in the thiol group interacting in a planar geometry with the surface. These results show the interest of theoretical calculation in the interpretation of corrosion measurements.

## Author contributions

AB proposed the idea and run the theoretical calculations. VM realized the experimental results. The article was written jointly by both of the authors.

### Conflict of interest statement

The authors declare that the research was conducted in the absence of any commercial or financial relationships that could be construed as a potential conflict of interest.
