# Supplementary material for: Corrosion Study of Mild Steel in Aqueous Sulfuric Acid Solution Using 4-Methyl-4H-1,2,4-Triazole-3-Thiol and 2-Mercaptonicotinic Acid—An Experimental and Theoretical Study
Source: Front Chem. 2017 Aug 24;5:61. doi: 10.3389/fchem.2017.00061 (PMC5609597; doi:10.3389/fchem.2017.00061)
Supplement: Supplementary file 1 [file DataSheet1.DOCX]

Supplementary Material

Corrosion study of mild steel in aqueous sulfuric acid solution using 4-methyl-4H-1,2,4-triazole-3-thiol and 2-mercaptonicotinic acid – an experimental and theoretical study

Valbonë Mehmeti, Avni Berisha*

*** Correspondence:** avni.berisha@uni-pr.edu

**Supplementary Figure 1.** Structures of: 2-mercaptonicotinic acid (2MA) and 4-methyl-4H-1,2,4-triazole-3-thiol in their nonprotonated (2MA and 4MT) and protonated (2MA-H+ and 4MT-H+) forms.


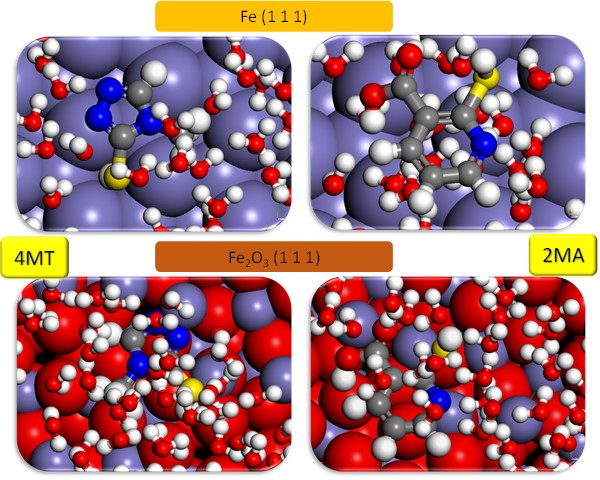


**Supplementary Figure 2.** Most appropriate configuration for the adsorption of 4MT and 2MA on Fe(111) and Fe_2_O_3_ (111) substrate in the presence of water attained by the adsorption locator module.


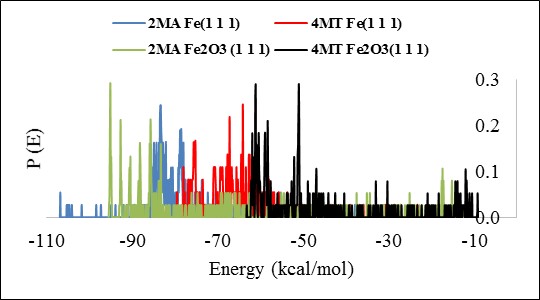


**Supplementary Figure 3.** The adsorption energy distribution of the adsorbate (2MA or 4MT) on Fe (111) and Fe_2_O_3_ (1 1 1) surface in presence of water molecules.


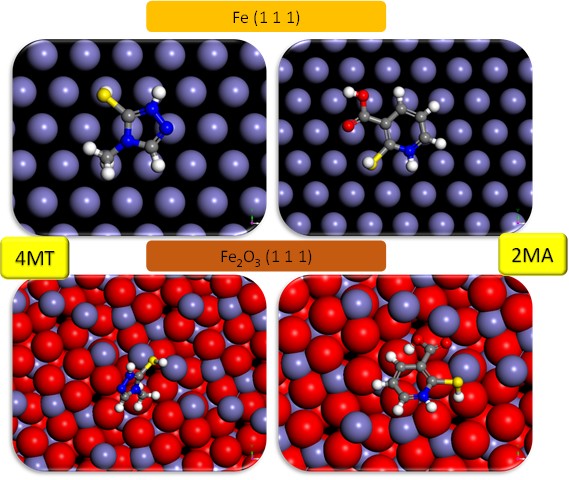


**Supplementary Figure 4.** Most appropriate configuration for the adsorption of 4MT and 2MA (protonated form) on Fe(111) and Fe_2_O_3_ (111) substrate attained by the adsorption locator module.


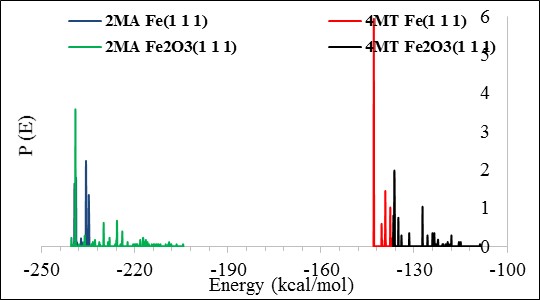


**Supplementary Figure 5.** The adsorption energy distribution of the protonated adsorbate molecules (2MA or 4MT) on Fe (111) and Fe_2_O_3_ (1 1 1) surface.

**Supplementary Table 1.** The molecular properties of the investigated compounds [2-mercaptonicotinic acid (2MA) and 4-methyl-4H-1,2,4-triazole-3-thiol in their nonprotonated (2MA and 4MT) and protonated (2MA-H^+^ and 4MT-H^+^)] using: DFT, B3LYP/6-31G (d,p). Results in vacuo and water. All energies are in eV.

| **Molecule** |  | **4MT(vacuum)** | **4MT(water)** | **2MA(vacuum)** | **2MA(water)** | **4MT-HJ+(vacuum)** | **4MT-H+(water)** | **2MA-H+(vacuum)** | **2MA-H+(water)** |
| --- | --- | --- | --- | --- | --- | --- | --- | --- | --- |
| E(HOMO) |  | -6.47 | -6.57 | -6.37 | -6.50 | -12.04 | -7.47 | -11.48 | -7.17 |
| E(LUMO) |  | -1.72 | 0.05 | 0.02 | 1.77 | -5.73 | -0.98 | -6.89 | -2.38 |
| ∆E |  | -4.75 | -6.62 | -6.39 | -8.27 | -6.31 | -6.49 | -4.59 | -4.79 |
| μ |  | 1.06 | 6.43 | 4.92 | 4.92 | 2.42 | 2.90 | 4.54 | 6.41 |
| IP |  | 6.47 | 6.57 | 6.37 | 6.50 | 12.04 | 7.47 | 6.50 | 7.17 |
| EA |  | 1.72 | -0.05 | -0.02 | -1.77 | 5.73 | 0.98 | -1.77 | 2.38 |
| χ |  | 4.10 | 3.26 | 3.18 | 2.37 | 8.89 | 4.23 | 2.37 | 4.78 |
| η |  | 2.38 | 3.31 | 3.20 | 4.14 | 3.16 | 3.25 | 4.14 | 2.40 |
| σ |  | 0.42 | 0.30 | 0.31 | 0.24 | 0.32 | 0.31 | 0.24 | 0.42 |
| ω |  | 0.24 | 6.25 | 3.79 | 2.93 | 0.93 | 1.30 | 4.49 | 8.58 |
